# The Role of the Immune System in the Pathogenesis of Diabetic Complications

**DOI:** 10.3389/fendo.2014.00126

**Published:** 2014-07-30

**Authors:** Gabriel Virella, Maria F. Lopes-Virella

**Affiliations:** ^1^Medical University of South Carolina, Charleston, SC, USA; ^2^Ralph E. Johnson VA Medical Center, Charleston, SC, USA

**Keywords:** immune system, pathogenesis, diabetic complications, diabetes complications, editorial

The main causes of morbidity and mortality in diabetes are macrovascular and microvascular complications. The pathogenesis of these complications is multifactorial, but there is strong evidence implicating chronic, smoldering, and inflammation as a main pathogenic event in the development of diabetic complications (1). Although the mechanisms responsible for vascular inflammation in diabetes are similar to those involved in vascular disease in non-diabetics (2), chronic hyperglycemia and dysregulated immune responses in diabetes are responsible for the activation of inflammatory circuits, inducing oxidative stress and promoting insulin resistance (1, 3), thus creating conditions that lead to the development of diabetes and diabetic complications. Changes in gene expression associated with diabetes like increased ICAM-1 expression (4) may also play a role in inducing inflammation and the development of diabetic complications.

Increased expression of adhesion molecules is associated with the development of diabetic complications. As discussed by Gu et al. (5), over-expression of ICAM-1, may be one of the key events in the development of nephropathy, as reflected by significant correlations between ICAM-1 levels and the development of proteinuria. Although the increased expression of ICAM-1 is not exclusive of diabetes, a linkage of the ICAM-1 gene to diabetes and diabetic nephropathy (5) suggests a selective involvement of this particular inflammatory event in both type 1 and type 2 diabetes. Clinically, ICAM-1 and other adhesion molecules like E-selectin can predict development of diabetic nephropathy and perhaps other complications in type 1 diabetes (5). Therapeutically, experimental data suggest that inhibiting ICAM-1 gene expression may prevent or slow down the development of diabetic nephropathy (5). As uncontrolled chronic inflammation progresses, kidney fibrosis develops, leading to end-stage kidney disease. Recruitment and activation of macrophages and of CD4^+^ T cells after initial tissue injury precede and have a critical role in the development of fibrosis, thus linking inflammation to the development of renal fibrosis (6). Several drugs used in the treatment of renal insufficiency have anti-inflammatory properties. However, the use of anti-inflammatory agents has not been effective in the treatment of diabetic nephropathy (6), likely because, once the fibrotic process becomes irreversible, the value of such interventions is limited.

Oxidation and other forms of modification of lipids and lipoproteins have emerged as a major pathogenic factor in atherosclerosis. Modified lipoproteins deliver pro-inflammatory signals that activate innate and adaptive immune responses and disturb the integrity of the microvasculature (3). In diabetes, the combination of hyperglycemia and increased oxidative stress results in enhanced LDL modification. Advanced glycation end-products (AGE)-modified LDL plays an important pathogenic role through its interactions with RAGE and angiotensin receptors (3). Oxidized LDL activates T cells, leading to enhanced inflammation through the release of macrophage-activating mediators (2). The adaptive humoral autoimmune response to modified forms of LDL is well characterized and strong evidence exists linking the formation of immune complexes (IC) involving modified forms of LDL and the corresponding autoantibodies with the development of diabetic complications. High levels of oxidized LDL in IC strongly predict the progression of atherosclerosis in patients with type 1 diabetes, while high levels of malondialdehyde-modified LDL in IC indicate strong risk for acute cardiovascular events in patients with type 2 diabetes (7).

Modified LDL molecules express a variety of immunogenic epitopes. The most immunogenic and better characterized are modified lysine epitopes, but oxidized phospholipids are also exposed to the immune system (2). Phosphorylcholine is a particularly interesting epitope because the resulting antibodies appear to protect against the development of atherosclerosis (2). Because phospholipid autoantibodies are usually of the IgM isotype, their protective effect could be a result of the reduced inflammatory potential of IgM IC, which cannot activate phagocytic cells through Fc receptors. Therefore, the end result of the humoral immune response to modified LDL could depend on which antibodies predominate: the strongly pro-inflammatory IgG antibodies or the non-inflammatory IgM antibodies.

There is a large diversity of autoantibodies, besides those directed to modified LDL and phospholipids that are believed to play a pathogenic role in diabetes. To that long list, Zimmering et al. added anti-neurotrophic antibodies, which seem to be involved in the pathogenesis of open-angle glaucoma and/or dementia in adult diabetics (4).

There is great interest in defining biomarkers predictive of diabetic complications. Among the many biomarkers that have been proposed, the blood levels of ICAM-1 (5), the levels of modified LDL in circulating IC (7), and the levels of fibroblast growth factor (8) are discussed in this e-book. While ICAM-1 levels and its polymorphisms appear linked to the development of nephropathy, the levels of modified LDL in IC and the levels of fibroblast growth factor have predictive value for cardiovascular disease in type 1 and type 2 diabetes. The results are quite strong since they were based on data obtained on a large number of patients.

As evidence supporting the pathogenic role of the adaptive immune response in diabetes accumulates, there has been a surge in the investigation of down-regulatory mechanisms that could be therapeutically exploited. The role of T regulatory (Treg) cells has been the object of considerable attention. Data generated in animal models suggest that Treg cells play a critical role in controlling the development of diabetes, both in type 1 and type 2 models (9). Also in animal models, there is data suggesting that administration of CD3 monoclonal antibodies permanently reverses diabetes in NOD mice. In humans, the results have not been so spectacular, but in type 1 diabetic patients CD3 antibody administration seems to preserve islet cell function for 1–5 years (9). Although human trials have never replicated the animal model data, the data remain very appealing.

A second alternative, also suggested by data in animal models, is to enhance the regulatory effect on incretin hormones, particularly glucagon-like peptide-1 (GLP-1) whose levels or effects can be enhanced by the administration of GLP-1 receptor agonists, or by inhibitors of dipeptidyl peptidase (DDP)-4, and enzyme that degrades GLP-1 (10). Data obtained in mice suggest that GLP-1 has modulatory effects, promoting the survival of Treg cells, and treatment with incretins suppresses the progression of atherosclerosis (10). Whether the administration of GLP-1R agonists or DDP-4 inhibitors may have similar effects in humans is a very interesting concept and worth investigating.

## Conflict of Interest Statement

The authors declare that the research was conducted in the absence of any commercial or financial relationships that could be construed as a potential conflict of interest.
